# Assessment of Left and Right Ventricular Diastolic and Systolic Functions Using Two-Dimensional Speckle-Tracking Echocardiography in Patients with Coronary Slow-Flow Phenomenon

**DOI:** 10.1371/journal.pone.0117979

**Published:** 2015-02-23

**Authors:** Yonghuai Wang, Chunyan Ma, Yan Zhang, Zhengyu Guan, Shuang Liu, Yuling Li, Jun Yang

**Affiliations:** 1 Department of Cardiovascular Ultrasound, The First Hospital of China Medical University, Shenyang, Liaoning, People’s Republic of China; 2 Department of Cardiology, The First Hospital of China Medical University, Shenyang, Liaoning, People’s Republic of China; Temple University, UNITED STATES

## Abstract

**Objective:**

Coronary slow-flow phenomenon (CSFP) is an angiographic diagnosis characterised by a low rate of flow of contrast agent in the normal or near-normal epicardial coronary arteries. Many of the patients with CSFP may experience recurrent acute coronary syndromes. However, current clinical practice tends to underestimate the impact of CSFP due to the yet unknown effect on the cardiac function. This study was performed to evaluate left ventricular (LV) and right ventricular (RV) diastolic and systolic functions, using two-dimensional (2D) longitudinal strain and strain rate, in patients with CSFP, and to determine the relationships between the thrombolysis in myocardial infarction (TIMI) frame count (TFC) and LV and RV diastolic and systolic functions.

**Methods:**

Sixty-three patients with CSFP and 45 age- and sex-matched controls without CSFP were enrolled in the study. Diagnosis of CSFP was made by TFC. LV and RV diastolic and systolic functions were assessed by 2D speckle-tracking echocardiography.

**Results:**

LV peak early diastolic longitudinal strain rate (LSRe) was lower in patients with CSFP than in controls (*P* = 0.01). LV peak systolic longitudinal strain (LS) and LV peak systolic longitudinal strain rate (LSRs) were lower in patients with CSFP than in controls (*P* = 0.004 and *P* = 0.03, respectively). There was no difference in LV ejection fraction. RV peak early diastolic longitudinal strain rate (RSRe) was lower in patients with CSFP than in controls (*P* = 0.03). There were no differences in RV peak systolic longitudinal strain (RS), RV peak systolic longitudinal strain rate (RSRs), or RV fractional area change among the groups. The mean TFC correlated negatively with LSRe and RSRe in patients with CSFP (*r* = −0.26, *P* = 0.04 and *r* = −0.32, *P* = 0.01, respectively).

**Conclusions:**

LV diastolic and systolic functions were impaired in patients with CSFP. CSFP also affected RV diastolic function, but not RV systolic function.

## Introduction

Coronary slow-flow phenomenon (CSFP), initially reported by Tambe et al. [1] in 1972, is a relatively rare angiographic finding observed in patients with normal or near-normal coronary arteries. CSFP is characterized by delayed opacification of coronary arteries during angiography. The frequency of CSFP is approximately 1% to 7% in patients undergoing coronary angiography [2], [3]. More than 80% of patients with CSFP often experience recurrent chest pain, almost 20% of whom require readmission following the same diagnosis [2], [3]. Although it has been well known to cardiologists for decades, the etiology and pathophysiologic mechanisms of the disease have not been well understood. Whether the left ventricular (LV) and/or right ventricular (RV) functions are affected by CSFP, and to what extent, is still not precisely known [4], [5]. Using tissue Doppler imaging (TDI), several studies found that LV diastolic and systolic functions were impaired and that CSFP did not affect RV function [6], [7]. However, TDI measurement depends on the direction of the Doppler angle. Two-dimensional speckle-tracking echocardiography (2D-STE) is a novel technique enabling more reliable and comprehensive assessment of myocardial function by obtaining the myocardial strain and strain rate in longitudinal, radial, and circumferential directions based on the tracking of speckles in grayscale 2D echocardiographic images [8], [9]. This modality negates the need for parallel alignment of the ultrasound beam to myocardial wall motion. 2D-STE has been found to be clinically useful in the assessment of cardiac systolic and diastolic functions [10–13]. Strain and strain rate, the parameters that represent the amount of myocardial deformation and the rate of deformation, can identify early subclinical changes in various abnormalities [14], [15]. In previous studies it has been shown that longitudinal functions deteriorate earlier than radial and circumferential functions in myocardial dysfunction [16], [17]. In the present study we aimed to evaluate LV and RV diastolic and systolic functions by measuring longitudinal strain and strain rate using 2D-STE in patients with CSFP. To our knowledge, to date this is the first study to explore this topic using this method.

## Subjects and Methods

### Study Population

This study was performed between June 2010 and October 2013. Over a 3-year period, of 4328 consecutive patients who underwent coronary arteriography in the cardiology department of our hospital, 108 patients with normal or near-normal (<40% stenosis) coronary arteries were enrolled in the study. We performed coronary angiography in these patients because they had typical angina and coronary risk factors. CSFP was defined as a corrected thrombolysis in myocardial infarction (TIMI) frame count (TFC) exceeding 27 in one or more vessels based on the recommendations of Gibson et al. [18]. Among the recruited patients, those with CSFP were allocated to the CSFP group. All patients were in normal sinus rhythm. Patients having the following features were excluded from the study: coronary artery ectasia, proximal lumen diameter of less than 3 mm, heart failure, valvular dysfunction, ventricular preexcitation, atrioventricular conduction abnormalities, bundle branch block, atrial fibrillation, paced rhythm, restrictive, hypertrophic, or dilated cardiomyopathies, congenital heart disease, previous history of myocardial infarction, uncontrolled hypertension, hyperthyroidism, hypothyroidism, malignancy, autoimmune disease, infection, pulmonary, hepatic, renal, and hematologic disorders, and poor echocardiographic image. Moreover, negative results on an exercise test were required to distinguish CSFP from syndrome X [19]. Any hemodynamic changes that might affect the TFC during coronary angiography were also excluded from the study. The remaining patients with normal coronary flow were allocated to the control group, with the same exclusion criteria during the study period. A total of 63 patients (26 females, 37 males; mean age 56±8 years) with CSFP and 45 age- and sex-matched control subjects (26 females, 19 males; mean age 55±8 years) were enrolled. All examinations were performed by investigators who had no information about the patients’ clinical status. All concomitant medications were stopped 48 hours prior to the procedure. Written informed consent was obtained from all participants, and the study was approved by the China Medical University Ethics Committee.

### Coronary Angiography and TFC

Coronary angiography was performed by the femoral approach using the standard Judkins technique. Coronary arteries were demonstrated in the left and right oblique planes and on cranial and caudal angulations. The same contrast medium was used in all patients. All injections were performed manually in all patients and control subjects. Coronary flow rates of all subjects were documented using the TFC. The TFC was determined for each major coronary artery of each subject included in the study, according to the method first described by Gibson et al. [18]. The number of frames, recorded at 30 frames per second, required for contrast material to reach a distal coronary landmark from the second it first appeared in the ostium of the related artery was counted and recorded. The distal coronary landmarks used for analysis were the distal bifurcation at the apex of the left anterior descending coronary artery (LAD) (the moustache, pitchfork, or whale’s tail), the distal bifurcation of the major obtuse marginal or the main left circumflex coronary artery (LCx), whichever was larger, and the site of origin of the first branch at the crux or its posterolateral extension for the right coronary artery (RCA). Since the LAD is usually longer than the LCx and RCA, the TFC for the LAD is often higher. Thus to obtain the corrected TFC of LAD (cLAD), the count was divided by 1.7. The TFCs of LAD and LCx were assessed in the right anterior oblique projection with caudal angulation, and that of RCA in the left anterior oblique projection with cranial angulation. The mean TFC for each subject was calculated by adding the TFC for RCA, LCx, and cLAD, and dividing the obtained value by 3. The TFCs were undertaken by two separate cardiologists. A third observer resolved any disagreement [6], [20].

### Echocardiography

Echocardiographic examination was performed within 72 hours after the coronary angiography. By using a Vivid 7 Dimension ultrasound system (GE Healthcare, Waukesha, WI, USA) with a 2- to 4-MHz phased-array probe, images were acquired in the left decubitus position during normal respiration. Right and left heart images and measurements were acquired from standard views according to the recommendations of the American Society of Echocardiography by two experienced cardiologists who were blinded to any clinical data [21]. At least three consecutive cardiac cycles were recorded.

### Conventional Two-Dimensional Echocardiography and Doppler Echocardiography

Left atrial anteroposterior dimension was measured during LV end-systole immediately before mitral valve opening from the parasternal long-axis view. LV end-diastolic dimension, interventricular septal thickness, and posterior wall thickness were measured during LV end-diastole immediately before aortic valve opening from the parasternal long-axis view. LV ejection fraction (LVEF), the index of global LV systolic function, was computed from apical two- and four-chamber views. LVEF was measured using the biplane modified Simpson method. Right atrium long-axis and minor-axis dimension and RV end-systolic area (RVESA) were measured during RV end-systole immediately before tricuspid valve opening in the apical four-chamber view. RV basal cavity diameter, RV mid cavity diameter, RV longitudinal diameter and RV end-diastolic area (RVEDA) were measured during RV end-diastole in the apical four-chamber view. The RV fractional area change (RVFAC), the index of global RV systolic function, is calculated as follows: RVFAC (%) = (RVEDA − RVESA)/RVEDA [21].

Pulsed-wave Doppler was performed in the apical four-chamber view to obtain mitral inflow velocities for the assessment of LV filling. Early diastolic mitral inflow velocity (mitral E) and late diastolic mitral inflow velocity (mitral A) were recorded. Pulsed-wave TDI images were acquired by activating the TDI functions of the echocardiography unit. From the apical four-chamber view, the sample volume should be positioned at the septal and lateral corner of the mitral annulus to record the longitudinal excursion of the mitral annulus. Primary measurements included the systolic (S’), early diastolic (E’), and late diastolic (A’) velocities. The tissue Doppler average signal of the septal and lateral sides of the mitral annulus was acquired to assess LV global function. The following measurements from the mitral annulus were made from the TDI recordings: mitral S’, E’, A’, E/A, E/ E’. Similar to Doppler-derived LV analysis, early diastolic tricuspid inflow velocity (tricuspid E) and late diastolic tricuspid inflow velocity (tricuspid A) were recorded. The following measurements from the lateral corner of the tricuspid annulus were made: tricuspid S’, E’, A’, E/A, E/ E’.

### Two-Dimensional Speckle-Tracking Echocardiography

Dynamic 2D ultrasound images of three cardiac cycles from apical two-, three-, and four-chamber views were acquired using conventional ultrasound, with a frame rate of 57 to 72 frames per second. To measure strain and strain rate, the image analysis was performed off-line using customized software within the EchoPAC work station (GE Healthcare). The endocardial boundary of the left ventricle was delineated manually, after which the software automatically drew the epicardial boundary. The widths of the regions of interest were adjusted manually to match the actual endocardial and epicardial boundaries. Automatic frame-by-frame tracking of speckle patterns during the cardiac cycle yielded a measure of strain and strain rate at any part of the myocardium. The LV myocardium was divided into six segments in each apical view, and each segment was individually analyzed. Subjects with inadequate tracking of more than one segment in at least one apical view were excluded from the study. By averaging all LV segmental values in all views, LV peak systolic longitudinal strain (LS) and strain rate (LSRs), LV peak early diastolic longitudinal strain rate (LSRe), and LV peak late diastolic longitudinal strain rate (LSRa) were calculated and obtained. Similar to STE-derived LV analysis, the longitudinal strain and strain rate of the basal, middle, and apical portions of RV free wall were obtained in apical four-chamber view. By averaging these segmental values, RV peak systolic longitudinal strain (RS) and strain rate (RSRs), RV peak early diastolic longitudinal strain rate (RSRe), and RV peak late diastolic longitudinal strain rate (RSRa) were calculated. The peak systolic longitudinal strain and strain rate represent ventricular longitudinal systolic function; the peak early diastolic longitudinal strain rate and peak late diastolic longitudinal strain rate represent ventricular longitudinal diastolic function.

### Statistical Analysis

Statistical analysis was performed using the SPSS 17.0 software package. Continuous data were expressed as mean ± standard deviation, and categorical variables as frequency and percentage. The Shapiro-Wilk test was used to evaluate whether the distribution of variables was normal. The Student *t*-test was used to compare normally distributed continuous variables. Categorical variables were compared using the chi-square test. For all parameters, a value of *P*<0.05 (two-tailed) was considered statistically significant.

## Results

One patient with CSFP and one control were excluded because of inadequate tracking quality of more than one segment in at least one apical view during the analysis of images by 2D-STE. In the remaining 106 subjects (62 CSFP patients and 44 control subjects), there were potentially 1,862 segments that could be tracked to obtain strain measurements. Of these, 46 segments (2.43%) were excluded from analysis because of inadequate tracking quality.

### Baseline Characteristics and Angiographic Findings of the Study Population

The baseline characteristics and angiographic findings of the CSFP and control groups are shown in Table 1. There was no difference in baseline characteristics among the groups. Patients with CSFP had significantly higher values of TFC for cLAD (42.6 ± 15.8 vs. 21.8 ± 3.1, *P*<0.001), LCx (36.9 ± 17.1 vs. 21.6 ± 3.9, *P*<0.001), and RCA (36.4 ± 19.3 vs. 21.2 ± 4.1, *P*<0.001), as well as mean TFC (38.6 ± 13.5 vs. 21.5 ± 2.3, *P*<0.001) than those with normal coronary flow. The LAD, LCx, and RCA were involved in 89%, 70%, and 56% of the patients, respectively. There was one-, two-, and three-vessel involvement in 24%, 36%, and 40% of the patients, respectively.

**Table 1:** The Baseline Characteristics and Angiographic Findings of the Study Population.

Variable	CSFP (n = 62)	Controls (n = 44)	*P*-value
Age (years)	56.7±8.6	55.5±8.2	0.49
Male sex (%)	36 (58%)	19 (43%)	0.13
Heart rate (beat/min)	66.0±9.2	68.8±7.6	0.10
Disease course (month)	43.5±55.5	43.6±53.7	1.00
Systolic blood pressure (mm Hg)	125.3±11.8	127.6±14.3	0.39
Diastolic blood pressure (mm Hg)	75.1±8.9	76.9±10.1	0.34
Hypertension (HTN) (%)	27 (44%)	18 (41%)	0.73
Diabetes mellitus (DM) (%)	4 (6%)	1 (2%)	0.31
Fasting blood glucose (mmol/L)	5.68±1.08	5.35±0.92	0.12
LDL cholesterol (mmol/L)	2.87±0.81	2.71±0.86	0.31
Triglycerides (mmol/L)	1.41±0.69	1.38±0.86	0.81
HDL cholesterol (mmol/L)	1.09±0.22	1.19±0.34	0.07
Total cholesterol (mmol/L)	4.40±0.94	4.34±0.91	0.76
Medications:			
ACEI/ARB (%)	26 (42%)	17 (38%)	0.61
Beta-blockers (%)	35 (56%)	22 (49%)	0.42
Calcium channel blocker (%)	7 (11%)	9 (21%)	0.15
Statin (%)	51 (83%)	33 (74%)	0.19
Nitrates (%)	51 (82%)	30 (68%)	0.08
TFC:			
cLAD	42.6±15.8	21.8±3.1	< 0.001
LCx	36.9±17.1	21.6±3.9	< 0.001
RCA	36.4±19.3	21.2±4.1	< 0.001
Mean	38.6±13.5	21.5±2.3	< 0.001

Values shown are Mean ± SD or percentage. TFC, thrombolysis in myocardial infarction frame count; LAD, left anterior descending coronary artery; LCx, left circumflex coronary artery; RCA, right coronary artery; LDL, low-density lipoprotein; HDL, high-density lipoprotein; ACEI, angiotensin-converting enzyme inhibitor; ARB, angiotensin II receptor blocker.

### LV and RV Echocardiography Measurements of the Study Population

The conventional 2D echocardiography and Doppler echocardiography parameters of the LV and RV are compared in Table 2. The results of 2D longitudinal strain and strain-rate measurements of LV and RV are displayed in Table 3.

**Table 2:** Conventional Two-Dimensional Echocardiography and Doppler Echocardiography Measures.

Parameters	CSFP (n = 62)	Controls (n = 44)	*P*-value
LA anteroposterior diameter (mm)	35.60±3.98	34.18±3.94	0.07
LV end-diastolic diameter (mm)	47.29±4.21	47.00±3.82	0.72
Interventricular septum (mm)	8.56±1.28	8.49±1.25	0.79
LV posterior wall (mm)	8.11±1.08	7.84±1.02	0.20
LV end-diastolic volume (ml)	84.98±16.69	81.30±15.74	0.26
LV end-systolic volume (ml)	31.38±7.21	29.17±7.42	0.13
LV ejection fraction (%)	62.90±3.84	64.26±4.42	0.10
Mitral E (m/s)	0.67±0.17	0.74±0.17	0.02
Mitral A (m/s)	0.72±0.13	0.68±0.16	0.17
Deceleration time of mitral E (ms)	176.84±27.35	183.49±29.45	0.23
Mitral E/A	0.96±0.30	1.15±0.35	0.003
Mitral S’ (cm/s)	8.76±1.50	8.98±3.96	0.69
Mitral E’ (cm/s)	7.94±2.04	8.44±2.42	0.24
Mitral A’ (cm/s)	9.17±1.40	9.13±1.52	0.88
Mitral E/ E’	8.68±2.54	9.21±2.40	0.28
RV basal cavity diameter (mm)	26.18±4.19	26.78±3.55	0.43
RV mid cavity diameter (mm)	27.57±4.99	26.69±4.26	0.34
RV longitudinal diameter (mm)	60.65±7.31	59.00±5.80	0.21
RA long axis dimension (mm)	46.55±4.27	44.87±5.39	0.08
RA minor axis dimension (mm)	36.02±5.20	36.51±4.34	0.60
RV end-diastolic area (cm^2^)	13.27±2.64	12.69±2.87	0.28
RV end-systolic area (cm^2^)	5.06±1.48	4.78±1.23	0.30
RV fractional area change (%)	62.05±6.62	62.13±4.70	0.94
Tricuspid E (m/s)	0.59±0.11	0.58±0.12	0.88
Tricuspid A (m/s)	0.46±0.11	0.43±0.09	0.11
Tricuspid E/A	1.33±0.31	1.41±0.36	0.19
Tricuspid S’ (cm/s)	12.18±2.10	12.98±2.70	0.09
Tricuspid E’ (cm/s)	8.47±2.07	9.80±2.46	0.003
Tricuspid A’ (cm/s)	12.24±3.10	13.07±3.24	0.19
Tricuspid E/ E’	7.36±2.35	6.27±1.82	0.01

Values shown are Mean ± SD. LA, left atrium; LV, left ventricular; RA, right atrium; RV, right ventricular; E, early diastolic flow velocity; A, late diastolic flow velocity; S’, systolic annular velocity; E’, early diastolic annular velocity; A’, late diastolic annular velocity.

**Table 3:** Longitudinal Strain and Strain Rate Measurements by 2D STE.

Parameters	CSFP (n = 62)	Controls (n = 44)	*P*-value
Left ventricular:			
Peak systolic longitudinal strain (%)	-19.25±2.26	-20.62±2.47	0.004
Peak systolic longitudinal strain rate (1/s)	-1.23±0.22	-1.32±0.20	0.03
Peak early diastolic longitudinal strain rate (1/s)	1.52±0.31	1.74±0.34	0.001
Peak late diastolic longitudinal strain rate (1/s)	1.27±0.26	1.28±0.25	0.79
Right ventricular:			
Peak systolic longitudinal strain (%)	-26.15±6.07	-27.02±6.65	0.49
Peak systolic longitudinal strain rate (1/s)	-1.95±0.47	-1.92±0.51	0.78
Peak early diastolic longitudinal strain rate (1/s)	1.86±0.61	2.12±0.56	0.03
Peak late diastolic longitudinal strain rate (1/s)	1.51±0.53	1.54±0.55	0.81

Values shown are Mean ± SD.

**Fig 1:**
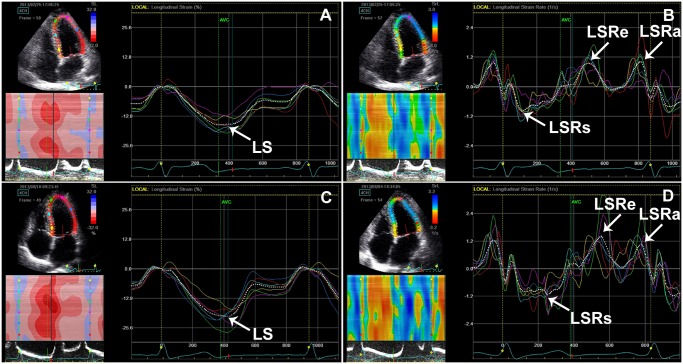
LV longitudinal strain and strain rate in patients with CSFP (A, B) and control subjects (C, D). Note the reduction of LS, LSRs and LSRe in patients with CSFP compared to control subjects. LS, LV peak systolic longitudinal strain; LSRs, LV peak systolic longitudinal strain rate; LSRe, LV peak early diastolic longitudinal strain rate; LSRa, LV peak late diastolic longitudinal strain rate.

**Fig 2:**
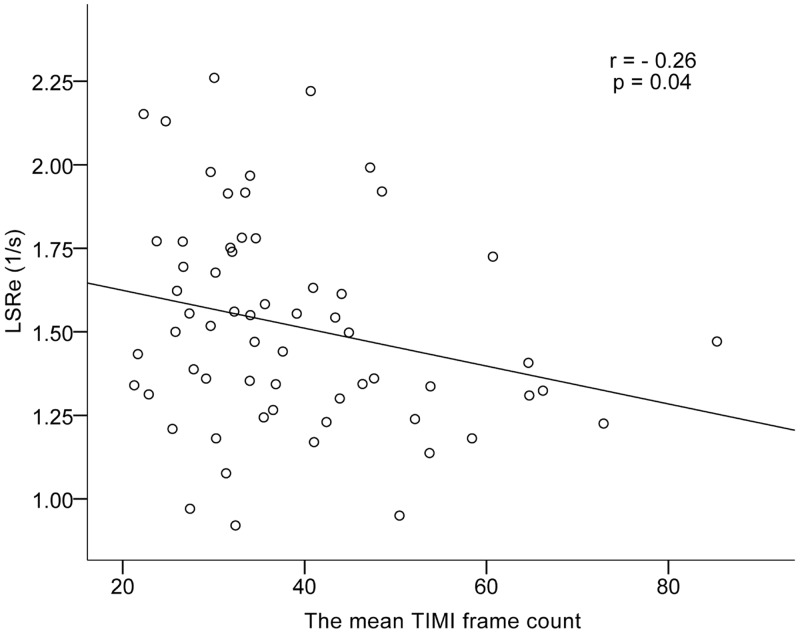
Scatter plot demonstrating correlation between the mean TFC with LSRe. TFC, the thrombolysis in myocardial infarction (TIMI) frame count; LSRe, LV peak early diastolic longitudinal strain rate.

Mitral E/A was significantly decreased in the patients with CSFP (*P* = 0.003). LSRe was significantly lower in the patients with CSFP than in control subjects (*P* = 0.01) (Fig. 1). LS and LSRs were significantly lower in the patients with CSFP than in control subjects (*P* = 0.004 and *P* = 0.03) (Fig. 1). There was no difference in LVEF between groups. The mean TFC correlated negatively with LSRe (*r* = −0.26, *P* = 0.04) (Fig. 2).

**Fig 3:**
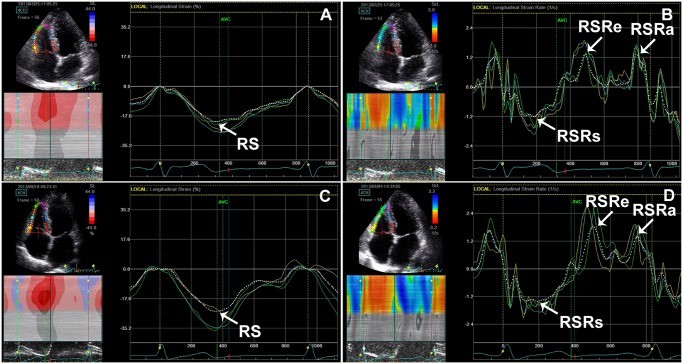
RV longitudinal strain and strain rate in patients with CSFP (A, B) and control subjects (C, D). Note the reduction of RSRe in patients with CSFP compared to control subjects. RS, RV peak systolic longitudinal strain; RSRs, RV peak systolic longitudinal strain rate; RSRe, RV peak early diastolic longitudinal strain rate; RSRa, RV peak late diastolic longitudinal strain rate.

**Fig 4:**
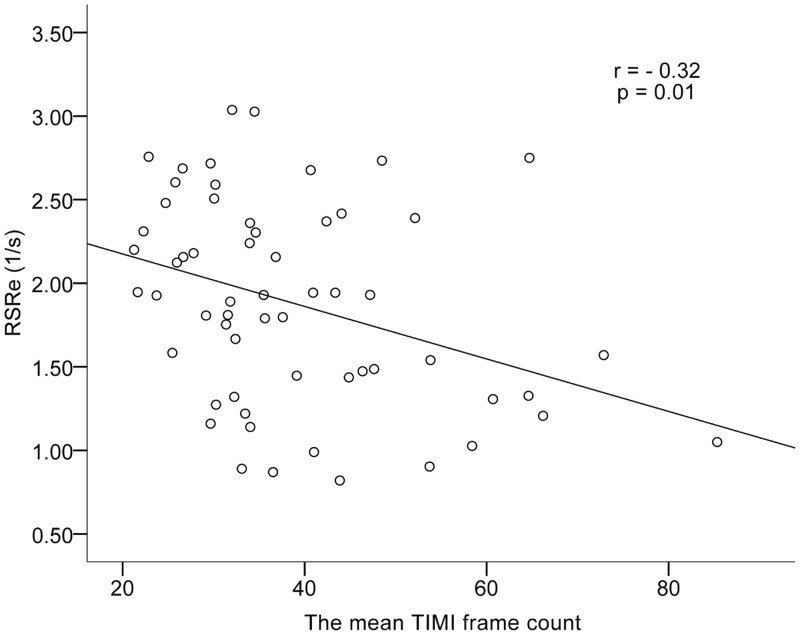
Scatter plot demonstrating correlation between the mean TFC with RSRe. TFC, the thrombolysis in myocardial infarction (TIMI) frame count; RSRe, RV peak early diastolic longitudinal strain rate.

Tricuspid E/ E’ of patients with CSFP was found to be higher than that of controls (*P* = 0.01). RSRe was significantly lower in the patients with CSFP than in control subjects (*P* = 0.03) (Fig. 3). RS and RSRs in the CSFP and control groups were equal (Fig. 3). There was no difference between groups for RVFAC. The mean TFC correlated negatively with RSRe (*r* = −0.32, *P* = 0.01) (Fig. 4).

## Discussion

The main objective of this study was to evaluate LV and RV diastolic and systolic functions in patients with CSFP. The main findings of our study were as follows. (1) CSFP affected RV diastolic function, but not RV systolic function. (2) The degree of deterioration of LV and RV diastolic functions had close relationships with the mean TFC.

CSFP is an angiographic diagnosis characterized by a low rate of flow of contrast agent in the normal or near-normal epicardial coronary arteries. In our study we chose to use the simple, reproducible, and validated means of the TFC to evaluate coronary flow. Previous studies showed that TFC in arteries with CSFP is significantly increased [1], [18], [22].

CSFP mimics various clinical presentations such as unstable angina, acute myocardial infarction, and ventricular tachycardia [23]. The precise etiology and pathophysiologic mechanism of CSFP are not sufficiently clear. Previous studies have postulated several mechanisms, such as endothelial dysfunction, microvascular dysfunction, early-stage coronary atherosclerosis, rheologic abnormalities, a systemic inflammatory state, and metabolic perturbations [22], [24–27]. Several investigators reported that fibromuscular hyperplasia, medial hypertrophy, myointimal proliferation and endothelial edema, thickening, and degeneration in the coronary microvessels were observed in biopsy samples of CSFP patients [22], [28]. Occlusive disease of small coronary arteries has been suggested as a possible cause of CSFP [28].

This study measured 2D longitudinal strain and strain rate, and demonstrated that both the LV diastolic and systolic functions were impaired in patients with CSFP. Nevertheless, TDI in previous studies demonstrated disagreements on the effect on LV diastolic and systolic functions in patients with CSFP. Altunkas et al. [7] found that CSFP only affected LV isovolumetric relaxation time. Nurkalem et al. [17] showed that there is an impairment in LV systolic function in patients with CSFP. Baykan et al. [6] demonstrated that both LV systolic and diastolic functions were impaired in patients with CSFP. Tissue Doppler signal has angle dependency and may be influenced by global heart motion, such as translation, torsion, and rotation [29]. These weaknesses of TDI may be the reasons for the aforementioned inconsistent views about the effect on LV function in patients with CSFP. 2D-STE is an emerging technology that measures strain and strain rate by tracking speckles in 2D grayscale echocardiographic images [8], [9]. It is able to measure myocardial motion in any direction irrespective of the direction of the beam, and provides strain in all dimensions (longitudinal, radial, and circumferential). This objective, comprehensive, and noninvasive methodology can detect and assess myocardial diastolic and systolic performance. Abnormalities of strain and strain rate can be found early in the development of many pathophysiologic states, and thus provide a sensitive means for detecting myocardial dysfunction [15], [29].

Studies evaluating RV function in patients with CSFP in the published literature are limited. In our study we found that CSFP affected RV diastolic function, but not RV systolic function, on assessment of longitudinal strain and strain rate using 2D-STE. In contrast to the left ventricle, only RV diastolic function was affected in patients with CSFP. Obviously the degree of impairment of LV and RV functions in the patients with CSFP was different. A probable explanation may be the difference in anatomy, mechanics, and function between the LV and RV [30]. The RV has a triangular base with a thin crescentic free wall and is designed as a volume pump, ejecting into the lower-resistance pulmonary circulation. Although the cardiac output is the same for both ventricles, the muscle mass of the RV is about one-sixth that of the left ventricle, and it performs one-fourth of the stroke work [31–33]. The RV, with its thinner wall and lower operating pressures, has a systolic-diastolic coronary blood flow ratio that is much greater than that of the LV [34]. Moreover, the RV is capable of extracting increased amounts of oxygen during hemodynamic stress [35]. In the absence of severe RV hypertrophy, the RV myocardial blood flow occurs during both systole and diastole. On the other hand, diastolic function disorder has its earliest onset during myocardial ischemia [36]. Therefore we consider that CSFP may not be enough to deteriorate the RV systolic functions, owing to the better hemodynamic profile of the RV [30]. RV diastolic dysfunction adversely affects LV diastolic properties [37]. Therefore, RV has great importance in the prognosis of patients with LV dysfunction. This also emphasizes the importance of close follow-up of RV function of patients with CSFP.

Our study showed the negative correlation between the mean TIMI frame count with LV and RV diastolic functions. The myocardial diastolic performance markedly decreased as the mean TIMI frame count increased. This finding emphasizes the importance of close attention to the patients with a larger number of TIMI frame count. However, there was no association between the mean TIMI frame count with myocardial systolic performance. An explanation for these findings may be that diastolic dysfunction is more sensitive to the severity of inadequate supply of the myocardium [36].

In view of the deterioration of ventricular function in patients with CSFP, clinical intervention should be timely employed in these patients to improve symptoms and patients’ quality of life. The conventional antianginal therapy was once one of the common treatment modalities for patients with CSFP, but its value was limited. It has been shown that resting coronary microvascular resistances are abnormally high and the unique calcium T-channel blocker mibefradil influence functional obstruction in arteries <200 μm [38]. We will further test the effectiveness of mibefradil by comparing the therapeutic group and the control group in the future.

### Limitations

The sample size of the study population may not have been adequate. We are expanding the sample size and performing a follow-up of these patients.

## Conclusion

LV systolic and diastolic functions were impaired in patients with CSFP. CSFP also affected RV diastolic function, but not RV systolic function. The degree of deterioration of LV and RV diastolic functions had close relationships with the mean TFC.
